# Biogenic amines analysis and microbial contribution in traditional fermented food of Douchi

**DOI:** 10.1038/s41598-018-30456-z

**Published:** 2018-08-22

**Authors:** Lu Li, Liying Ruan, Anying Ji, Zhiyou Wen, Shouwen Chen, Ling Wang, Xuetuan Wei

**Affiliations:** 1 Key Laboratory of Environment Correlative Dietology (Ministry of Education), College of Food Science and Technology, Huazhong Agricultural University, Wuhan, 430070 China; 2 State Key Laboratory of Agricultural Microbiology, Huazhong Agricultural University, Wuhan, 430070 China; 3 Hubei Collaborative Innovation Center for Green Transformation of Bio-Resources, College of Life Sciences, Hubei University, Wuhan, 430062 China; 4 Department of Food Science and Human Nutrition, Iowa State University, 50011 Ames, IA USA

## Abstract

Biogenic amines (BAs) have been reported to threaten the Douchi safety, while the BAs formation mechanism and corresponding control method have not been clarified for Douchi. The present study aims to investigate the microbial contribution to BAs in Douchi, and to find the beneficial strain for BAs control. Firstly, the BAs profiles of 15 Douchi samples were analyzed, and common 6 kinds of BAs were detected from different samples. All the samples showed the total BAs contents within the safe dosage range, while the histamine concentrations in 2 samples and β-phenethylamine in 6 samples were above the toxic level. Then, the bacterial and fungal communities were investigated by high-throughput sequencing analysis, and *Bacillus* and *Candida* were identified as the dominant bacteria and fungi genus, respectively. Furthermore, nineteen strains were selected from the dominant species of Douchi samples, including 14 *Bacillus* strains, 2 *Staphylococcus* strains, 1 *Enterococcus* strain and 2 *Candida* strains, and their BAs formation and degradation abilities were evaluated. *B*. *subtilis* HB-1 and *S*. *pasteuri* JX-2 showed no BAs producing ability, and *B*. *subtilis* GD-4 and *Candida* sp. JX-3 exhibited high BAs degradation ability. Finally, fermented soybean model analysis further verified that *B*. *subtilis* HB-1 and *S*. *pasteuri* JX-2 could significantly reduce BAs. This study not only contributed to understanding the BAs formation mechanism in Douchi, but also provided potential candidates to control the BAs in fermented soybean products.

## Introduction

Biogenic amines (BAs) are organic nitrogenous compounds with low molecular weight, and they are formed during normal metabolic processes in various organisms^1^. BAs can be categorized into aliphatic (putrescine and cadaverine), aromatic (tyramine and β-phenethylamine) and heterocyclic amines (histamine and tryptamine) based on their chemical structures^2,3^. Recently, BAs have been broadly reported in various fermented foods, such as cheese, beer, wine and fermented soybeans, which shows potential harmful risk for human health^4–10^. BAs at low concentrations are essential for many physiological functions, while high concentrations of BAs can cause some deleterious effects^7,11^. Histamine can cause adverse neurological, gastrointestinal, circulatory and respiratory symptoms, such as headache, nausea, hot flushes, skin rashes and intestinal problems^12^. Tyramine, tryptamine and β-phenylethylamine have been confirmed to be implicated in hypertensive crises and dietary-induced migraine^13^. Therefore, evaluation and control of BAs are highly important to guarantee the safety of fermented food.

Douchi is a traditional fermented soybean product commonly consumed as flavouring agents in China, Japan, and other Asian countries^14–16^. The BAs have been reported as potential toxic substances in commercial Douchi^17–19^, thus evaluation and control of the BAs are valuable for the safety of Douchi. The BAs are mainly generated through decarboxylation of amino acids by various microbes, and putrescine, cadaverine, tyramine, β-phenethylamine, histamine and tryptamine were generated from ornithine, lysine, tyrosine, phenylethylamine, histidine and tryptophan, respectively^20^. Moreover, putrescine can also be formed through deimination of agmatine^21^. Different microbes may excrete different amino acid decarboxylases to produce specific BA, and some can also produce amine oxidases to degrade BAs^22–25^. Hence, the relations between BAs and microbes are complicated. Using the high BAs-degrading strains or the low BAs-producing strains as the starter cultures will be beneficial to control the Douchi BAs.

Generally, Douchi is produced by spontaneous fermentation with an uncontrolled manner, and various microbial communities are formed at different environment conditions^14–16^. The microbial diversities in commercial Douchi have been investigated using traditional culture method, denaturing gradient gel electrophoresis (DGGE) and high-throughput sequencing^16,26–28^, and the *Bacillus* species were identified as the dominant bacteria in Douchi^11,14,15,27^. However, the microbial contributions on BAs formation in Douchi have not been clarified, and the efficient starter cultures for BAs control have not developed. In this study, BAs contents and microbial communities in Douchi samples from different regions were investigated, and the BAs producing and degradation abilities of representative strains were evaluated to elucidate the microbial contribution and select beneficial candidate for BAs control.

## Materials and Methods

### Samples and media

Fifteen fresh Douchi samples were purchased from 15 different regions in China, named JXJJ (Jiangxi), GXWZ (Guangxi), GDYJ (Guangdong), SXHZ (Shanxi), YNKM (Yunnan), GZTR (Guizhou), HNLY (Hunan), HBYC (Hubei), CQ (Chongqing), GSLN (Gansu), SCCD (Sichuan), TJ (Tianjing), ZJJS (Zhejiang), JSXZ (Jiangsu) and HNLB (Henan). All samples were collected in three replicates, and stored at 4 °C for further analysis. The media used this study included LB medium (peptone 10 g/L, yeast extract 5 g/L, sodium chloride 10 g/L), MRS medium (peptone 10 g/L, beef extract 8 g/L, yeast extract 4 g/L, glucose 20 g/L, diammonium hydrogen citrate 2 g/L, sodium acetate 5 g/L, K_2_HPO_4_ 2 g/L, MgSO_4_ 0.2 g/L, MnSO_4_ 0.04 g/L, Tween 80 1 g/L, pH = 5.7), and SDB medium (peptone 10 g/L, glucose 20 g/L, pH = 5.6), and 1.5% agar was added to prepare solid medium.

### Illumina MiSeq sequencing

The total DNA was extracted from each Douchi sample by a SDS-based DNA extraction method previously described^29^. DNA quality was monitored by agarose gel electrophoresis, and stored at −20 °C for subsequent analysis. Primers of 515 F (GTGCCAGCMGCCGCGGTAA) and 806 R (GGACTACHVGGGTWTCTAAT) were designed according to the V4 region of bacterial 16S rRNA gene, and ITS1 (TCCGTAGGTGAACCTGCGG) and ITS2 (GCTGCGTTCTTCATCGATGC) were designed based on the ITS1 region of the fungal internal transcribed spacer (ITS).

The Miseq sequencing for bacteria and fungi followed the protocols described by Caporaso *et al*. and Kozich *et al*.^30,31^, respectively. The bacterial 16S rDNA and fungal ITS genes fragments were amplified using the total DNA, primers, and Thermo Scientific® Phusion High-Fidelity PCR Master Mix (New England Biolabs, UK), purified by the QIAquick PCR Purification Kit (QIAGEN, Germany). Then, purified amplicons were employed for DNA library construction using the TruSeq® DNA PCR-Free Sample Preparation Kit (Illumina). At last, DNA libraries were evaluated using Qubit@ 2.0 Fluorometer (Thermo Scientific) and real-time PCR, then sequenced on the MiSeq platform at Beijing Novogene Bioinformatics Technology Co., Ltd. All experiments were conducted in three replicates.

### Bioinformatics analysis

Raw sequences generated through MiSeq sequencing were merged using fast length adjustment of short reads (FLASH)^32^, and low-quality sequences were discarded using QIIME. Clean paired sequences retained for each sample were analyzed using the UPARSE pipeline to generate operational taxonomic units (OTUs) and select representative sequences at 97% similarity^33^. PyNAST alignment and ribosomal database project (RDP) assignment were carried out based on the latest Green genes database^34^. Resampling was performed according to the minimum sequence numbers across all samples before the downstream analysis. In addition, the community compositions were provided at different taxonomic levels.

### Isolation of strains from Douchi samples

Douchi samples (5 g) were added into 45 mL sterile water, rotated at 37 °C and 140 rpm for 40 min. The liquid mixtures were diluted, and spread onto the LB plates, MRS plates added with 20 g/L CaCO_3_ and SDB plates added with 50 mg/L rifampicin, respectively. The LB and SDB plates were cultured at 37 °C and 30 °C to isolate the dominant bacteria and fungus strains, respectively, and the MRS plates were incubated at 37 °C in a vacuum bag to select the lactic acid bacteria.

### Identification of strains

The bacterial genomic DNA was extracted by Gen-EluteTM Kit (Tiangen Biotech Co., Ltd, Beijing, China) following the manufacturer’s protocol, and the fungal genomic DNA was extracted using a SDS-based DNA extraction method previously described^29^. The 16S rDNA fragment was amplified through the universal primers of 27f (AGAGTTTGATCMTGGCTCAG) and 1492r (CTACGGCTACCTTGTTACGA), and the ITS fragment was amplified with the universal primers ITS1 (TCCGTAGGTGAACCTGCGG) and ITS4 (GCATATCAATAAGCGGA). The PCR protocol was set as: 95 °C, 5 min; 95 °C, 45 s, 55 °C, 1 min, 72 °C, 1 min, 32 cycles; 72 °C, 10 min; and 4 °C for 10 min. The PCR is conducted in the 25 μL reaction system consisting of 2 μL genomic template, 2.5 μL Easy-Taq Buffer, 2 μL primers, 2.5 μL dNTPs, 0.3 μL Easy-Taq enzyme and 15.7 μL nuclease-free water. The DNA fragments were sequenced by Tsingke Biological Technology Co., Ltd. (Wuhan, China), and the sequence identity was analyzed using the Blastn program (http://blast.ncbi.nlm.nih.gov/Blast.cgi).

### Evaluation of BAs production and degradation ability

The BAs production ability was assessed by culturing the strains in 5 mL LB (*Bacillus* and *Staphylococcus*), MRS (*Enterococcus*) or SDB (*Candida*) medium added with 1 g/L of histidine, tyrosine, tryptophan, phenylalanine, ornithine monohydrochloride, lysine or agmatine sulfate salt. After incubating for 48 h, the resulting BAs were determined. To measure the BAs-degrading ability, the cells were cultured and collected by centrifugation at 6000 × g for 5 min. After washing with 0.05 mol/L phosphate buffer (pH = 7), the cell pellets were diluted to OD600 = 0.4 in phosphate buffer (0.05 mol/L) containing 100 mg/L of histamine, tyramine, tryptamine, β-phenethylamine, putrescine and cadaverine, cultured for 48 h to detect the residual BAs, and the blank phosphate buffer without cell pellets was applied as the control. The BA-degradation rate was calculated according to the equation i.e., M = [(A − B)/A] × 100%, where M means the BAs degradation percentage, A and B indicate the initial and residual contents of BAs, respectively^20^.

### Fermented soybean product model analysis

The fermented soybean model was applied to compare the BAs-controlling capacities of different strains. The cells (OD600 = 2) were inoculated into the soaked soybeans (30 g) with the inoculum size of 5% (v/w), incubated for 7 days, and the total BAs contents were measured by HPLC. The soybeans were fermented by inoculating with *B*. *subtilis* HB-1, *S*. *pasteuri* JX-2, *B*. *subtilis* GD-4 and *Candida* sp. JX-3, respectively, and the soybeans added with equivalent sterile water was set as the control.

### BAs determination

The BAs contents were detected based on a previous report^20^. Briefly, 1.5 mL of 0.4 M HClO_4_ was added into 0.5 g solid sample or 0.5 mL liquid sample, extracted for 1 h. After centrifugation at 12,000 × g for 10 min, the supernatant (250 μL) was added with 25 μL of 2 M NaOH and 75 μL of saturated NaHCO_3_, then reacted with 500 μL of 5 mg/mL dansyl chloride at 50 °C for 45 min. After that, the reactant was mixed with 25 μL of 25% NH_4_OH and incubated at 50 °C for 15 min to remove the residual dansyl chloride. Then the mixture was adjusted to 1.5 mL with acetonitrile and centrifuged at 2500 × g for 5 min. The supernatant was filtered through a 0.22 μm membrane for HPLC (high-performance liquid chromatography) analysis, which was carried out using an Agilent 1260 HPLC system with an Agilent column Zorbax Eclipse XDB-C18 (4.6 mm × 250 mm, 5 μm) at 30 °C. The separation was achieved using a linear gradient of mobile phase A (acetonitrile) and B (H_2_O) at a flow rate of 1 mL/min. The solvent gradient was as follows: 50% A (0–3 min), 50–90% A (3–20 min), 90% A (20–29 min), 90–50% A (29–32 min), 50% A (32–35 min). The detection was carried out at 254 nm. Each BA in the samples was quantified with a calibration curve generating by analyzing standard BA solution. Typical chromatograms are shown in Figure S1.

### Statistical analysis

Alpha diversity including Chao 1, Shannon diversity indices and Goods coverage were subjected to statistical analysis using Qiime (Version 1.7.0). The community structure was statistically analyzed at different classification levels. The significance was analyzed by one-way ANOVA using the statistical software SPSS 20.0, and means were compared by Duncan’s multiple range test at 5%.

## Results and Discussion

### BAs contents in Douchi samples

BAs were widely detected in various soybean foods. Kim *et al*. detected six BAs in fermented soybean products, natto^4^, and Gong *et al*. reported that tryptamine, putrescine and cadaverine were detected in Douchi samples^19^. In this study, six common BAs with different contents were found among the Douchi samples (Table 1), including histamine (1.43–213.13 mg/kg), tyramine (1.37–64.33 mg/kg), tryptamine (0.86–99.31 mg/kg), β-phenethylamine (1.47–191.24 mg/kg), putrescine (2.65–171.00 mg/kg), and cadaverine (0.49–397.70 mg/kg), and the total BA contents ranged from 5.09 to 679.46 mg/kg. Generally, total BA contents in food are expected to be not more than 900 mg/kg^3,35,36^, and they may cause serious harm to human health when the total contents exceed 1000 mg/kg^24^. Our results indicated that no sample was above the harmful level (900 mg/kg) in the total content level, and as-detected samples were in the relative safe level on the whole. However, different BA showed different toxic level for human, thus each BA is needed to be further analyzed for comprehensive safety assessment.

**Table 1:** Contents of BAs in Douchi samples.

Douchi Sample	Histamine (mg/Kg)	Tyramine (mg/Kg)	Tryptamine (mg/Kg)	β-Phenethylamine (mg/Kg)	Putrescine (mg/Kg)	Cadaverine (mg/Kg)	Total (mg/Kg)
JXJJ	75.16 ± 3.41 c	54.80 ± 4.32 f	14.71 ± 1.24 d	191.24 ± 2.93 j	97.08 ± 1.27 j	246.47 ± 4.16 f	679.46 ± 17.33 j
GXWZ	1.43 ± 0.11 a	64.33 ± 1.85 h	ND	86.58 ± 0.91 i	45.62 ± 0.48 h	397.70 ± 4.35 g	595.66 ± 7.70 i
GDYJ	213.13 ± 2.37 d	20.81 ± 1.31 d	9.08 ± 1.79 bc	69.44 ± 3.40 h	171.00 ± 3.13 k	25.08 ± 0.90 e	508.54 ± 12.9 h
SXHZ	ND	45.33 ± 3.72 e	11.38 ± 0.48 cd	38.57 ± 1.38 f	71.66 ± 0.23 i	2.69 ± 0.03 abc	169.63 ± 5.84 g
YNKM	11.54 ± 0.47 b	59.94 ± 6.56 g	0.86 ± 0.18 a	69.11 ± 0.65 h	12.68 ± 0.33 d	5.62 ± 0.18 cd	159.75 ± 8.37 fg
GZTR	ND	2.09 ± 0.23 ab	99.31 ± 6.41 e	24.02 ± 2.05 e	19.25 ± 1.31 f	4.83 ± 0.57 bcd	149.50 ± 10.57 f
HNLY	11.84 ± 1.61 b	11.30 ± 2.39 c	ND	48.47 ± 4.79 g	15.09 ± 2.86 e	7.39 ± 0.91 d	94.09 ± 12.56 e
HBYC	ND	16.64 ± 2.94 d	5.89 ± 0.21 b	14.59 ± 0.54 d	8.59 ± 0.38 c	1.70 ± 0.16 ab	47.41 ± 4.23 d
CQ	ND	5.44 ± 0.38 b	ND	4.90 ± 0.46 ab	18.85 ± 0.05 f	0.54 ± 0.00 a	29.73 ± 0.89 c
GSLN	ND	ND	ND	ND	23.33 ± 0.30 g	1.93 ± 0.17 ab	25.26 ± 0.47 c
SCCD	ND	0.26 ± 0.09 a	ND	ND	21.46 ± 0.68 g	2.87 ± 0.14 abc	24.53 ± 0.91 c
TJ	ND	6.24 ± 0.84 b	ND	7.19 ± 2.40 bc	12.72 ± 1.24 d	ND	26.15 ± 4.48 c
ZJJS	ND	4.08 ± 0.13 ab	ND	8.79 ± 0.25 c	8.16 ± 0.41 c	ND	21.03 ± 0.79 bc
JSXZ	ND	1.37 ± 0.34 ab	ND	1.47 ± 0.24 a	5.49 ± 0.06 b	0.49 ± 0.20 a	8.82 ± 0.84 ab
HNLB	ND	2.44 ± 0.44 ab	ND	ND	2.65 ± 0.14 a	ND	5.09 ± 0.58 a

Data are presented as mean ± SDs of three replicates; ND means “not detected”. Different letters (a, b, c, etc.) indicate significantly different means at P < 0.05.

As shown in Table 1, histamine was detected in five Douchi samples, the histamine concentration of two samples were higher than the allowable limit (50 mg/kg) suggested by the US Food and Drug Administration^3,13^. And 100–800 mg/kg of tyramine and 30 mg/kg of β-phenylethylamine have been reported to be the toxic doses in food^4,10,37^. Although most of the Douchi samples contained tyramine, no sample showed the amount above the hazardous level (100 mg/kg). Tryptamine was detected in six samples, and their concentrations were under 100 mg/kg. Though no β-phenethylamine was detected in three samples, it was noteworthy that the concentrations of six products were higher than the toxic dose (30 mg/kg). While Gong *et al*. detected no histamine, tyramine and β-phenethylamine in Douchi samples^19^. The differences in distribution and content of BAs could be attributed to the variation of the raw materials, the microbiological compositions and the fermentation conditions.

Putrescine and cadaverine are regarded as two typical BA reported in soybean products including sufu, soy sauce, tempe, miso and natto^4,7^. Though putrescine was found in all Douchi samples, the concentrations were under 100 mg/kg in 14 Douchi samples. And the content of putrescine under 100 mg/kg has been usually attributed to natural occurrence of amine in soybean products^7^. Meanwhile, cadaverine was detected in twelve products, while the concentrations of cadaverine in two samples were higher than 200 mg/kg. The toxicity of putrescine and cadaverine is not obvious relatively, but they can interact with nitrite to form carcinogenic nitrosamine^38^.

### Microbial communities among the Douchi samples

As described above, BAs contents in Douchi samples showed relatively large variations, which was probably due to the different microbiological compositions. Therefore, it is important to analysis the microbial communities among the Douchi samples. By high-throughput sequencing, 3,514,240 16S rRNA (V3–V4 regions) and 3,402,964 ITS2 reads were generated from 45 sequencing samples. After quality filtering and chimera removal, 62,603 bacterial and 74,327 fungal sequences were obtained from each sample, with the average read length of 426 bases for bacteria and 211 bases for fungi. Community richness and diversity were assessed using four alpha-diversity metrics (observed OTU, Chao1, Shannon and Good’s sample coverage) (Table 2). Chao1 is an estimator of species richness, and Chao1 values indicated that all the Douchi samples possessed good sample richness. The Shannon diversity index, a measurement of overall diversity, indicated the diversified microbiota throughout the samples, and the Good’s coverage result, an estimator of completeness of sampling^39^, highlighted good overall sampling, with levels of 99%.

**Table 2:** Alpha-diversity metrics characterizing Douchi samples and inferred from the sequencing of 16S V3–V4 and ITS amplicons. Sequences were clustered at 97% identity.

Douchi sample	Bacteria				Fungi			
	Observed species	Chao1	Shannon	Good’s coverage (%)	Observed species	Chao1	Shannon	Good’s coverage (%)
JXJJ	503 ± 52 c	571.94 ± 106.20 bcd	4.20 ± 0.15 cd	99	115 ± 38 a	136.41 ± 57.77 a	1.06 ± 0.34 a	99
GXWZ	360 ± 76 abc	489.92 ± 165.10 abc	4.12 ± 0.16 bcd	99	121 ± 108 ab	153.31 ± 117.03 ab	2.08 ± 1.29 a	99
GDYJ	283 ± 80 abc	408.98 ± 108.89 abc	2.35 ± 0.28 abc	99	92 ± 52 a	115.29 ± 61.53 a	1.63 ± 1.10 a	99
SXHZ	274 ± 85 abc	400.36 ± 98.48 abc	3.60 ± 0.32 abcd	99	131 ± 20 ab	156.35 ± 27.29 ab	1.81 ± 0.35 a	99
YNKM	555 ± 196 cd	667.53 ± 231.33 cd	4.72 ± 0.73 d	99	126 ± 31 ab	149.72 ± 28.19 a	1.63 ± 1.35 a	99
GZTR	149 ± 23 a	191.62 ± 48.98 a	2.07 ± 0.22 ab	99	235 ± 103 cd	274.10 ± 110.50 bcd	3.44 ± 0.77 bc	99
HNLY	389 ± 47 abc	522.55 ± 84.83 abcd	1.75 ± 0.70 a	99	122 ± 82 ab	141.24 ± 105.69 a	1.65 ± 1.04 a	99
HBYC	196 ± 66 ab	269.26 ± 87.25 ab	2.42 ± 0.19 abc	99	246 ± 62 d	284.65 ± 99.82 cd	3.63 ± 0.39 c	99
CQ	549 ± 76 cd	625.56 ± 71.96 bcd	3.98 ± 1.90 bcd	99	174 ± 0 abcd	197.38 ± 0 abc	2.12 ± 0 a	99
GSLN	362 ± 47 abc	426.41 ± 44.12 abc	4.00 ± 0.25 bcd	99	220 ± 16 bcd	273.77 ± 26.88 bcd	3.49 ± 0.32 bc	99
SCCD	481 ± 422 bc	558.25 ± 535.29 bcd	5.10 ± 1.69 d	99	223 ± 0 bcd	349.04 ± 0 d	1.20 ± 0 a	99
TJ	471 ± 66 bc	581.94 ± 53.24 bcd	3.58 ± 0.51 abcd	99	98 ± 6 a	119.90 ± 13.96 a	2.26 ± 0.13 ab	99
ZJJS	789 ± 291 d	863.44 ± 275.59 d	4.71 ± 1.52 d	99	87 ± 4 a	103.00 ± 6.77 a	1.08 ± 0.32 a	99
JSXZ	440 ± 62 abc	562.34 ± 96.40 bcd	2.04 ± 0.95 ab	99	136 ± 50 abc	193.42 ± 44.81 abc	1.28 ± 1.00 a	99
HNLB	444 ± 96 abc	528.99 ± 67.78 abcd	3.04 ± 2.51 abcd	99	157 ± 50 abcd	185.22 ± 60.82 abc	1.65 ± 0.44 a	99

Data are presented as mean ± SDs of three replicates. Different letters (a, b, c, etc.) indicate significantly different means at P < 0.05.

**Figure 1:**
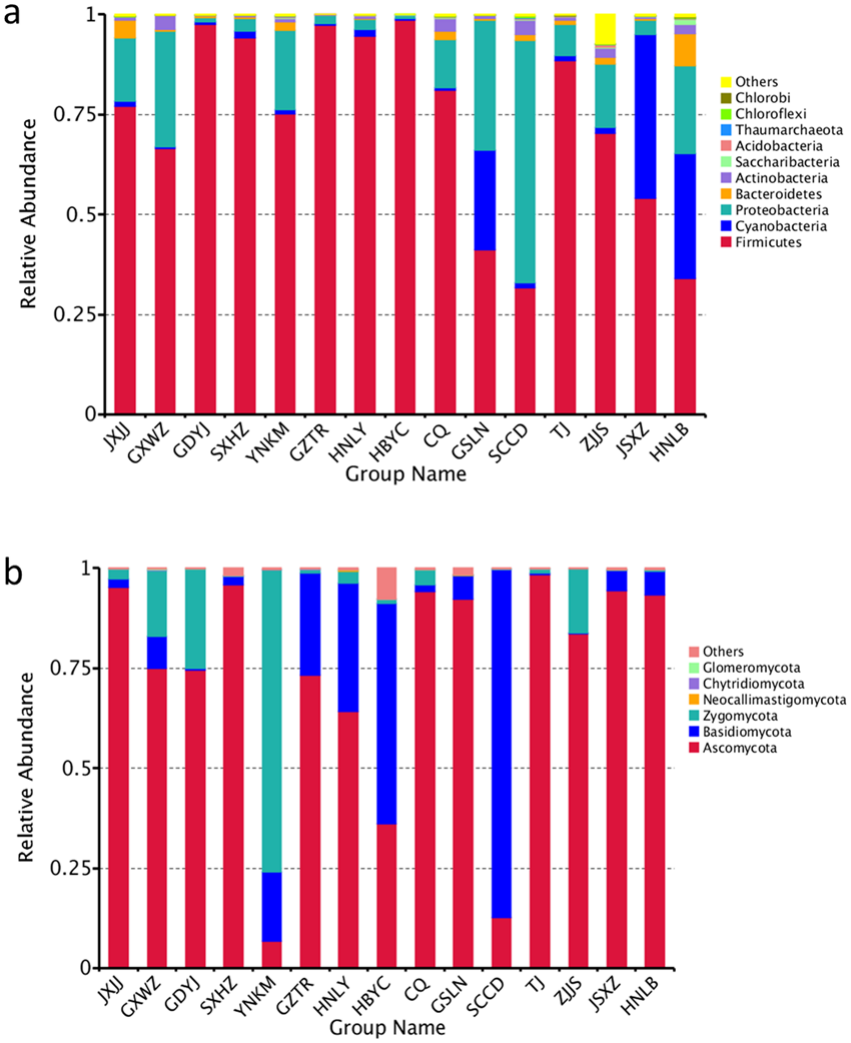
Microbial community in Douchi samples at the phylum level. (**a**) Bacteria, (**b**) Fungi.

As to bacteria, *Firmicutes* was the predominant phylum in most Douchi samples, while *Proteobacteria* was only dominant in the sample of SCCD (Fig. 1a). Yang *et al*. found *Firmicutes* and *Proteobacteria* were major phyla in Douchi fermentation^16^. At the genus level, *Bacillus* was the dominant bacterial genus in most Douchi samples (Fig. 1a), and it was also reported as the predominant genus in various fermented soybean products^40–42^. In addition, the genera of *Weissella*, *Enterococcus*, *Pantoea* and *Tetragenococcus* were also the major genera in some Douchi samples, which was consistent with previous reports^8,16,43,44^. In many fermented soybean products, *Bacillus* and *Tetragenococcus* played the crucial role in production of nutrients and flavors^26,45^.

**Figure 2:**
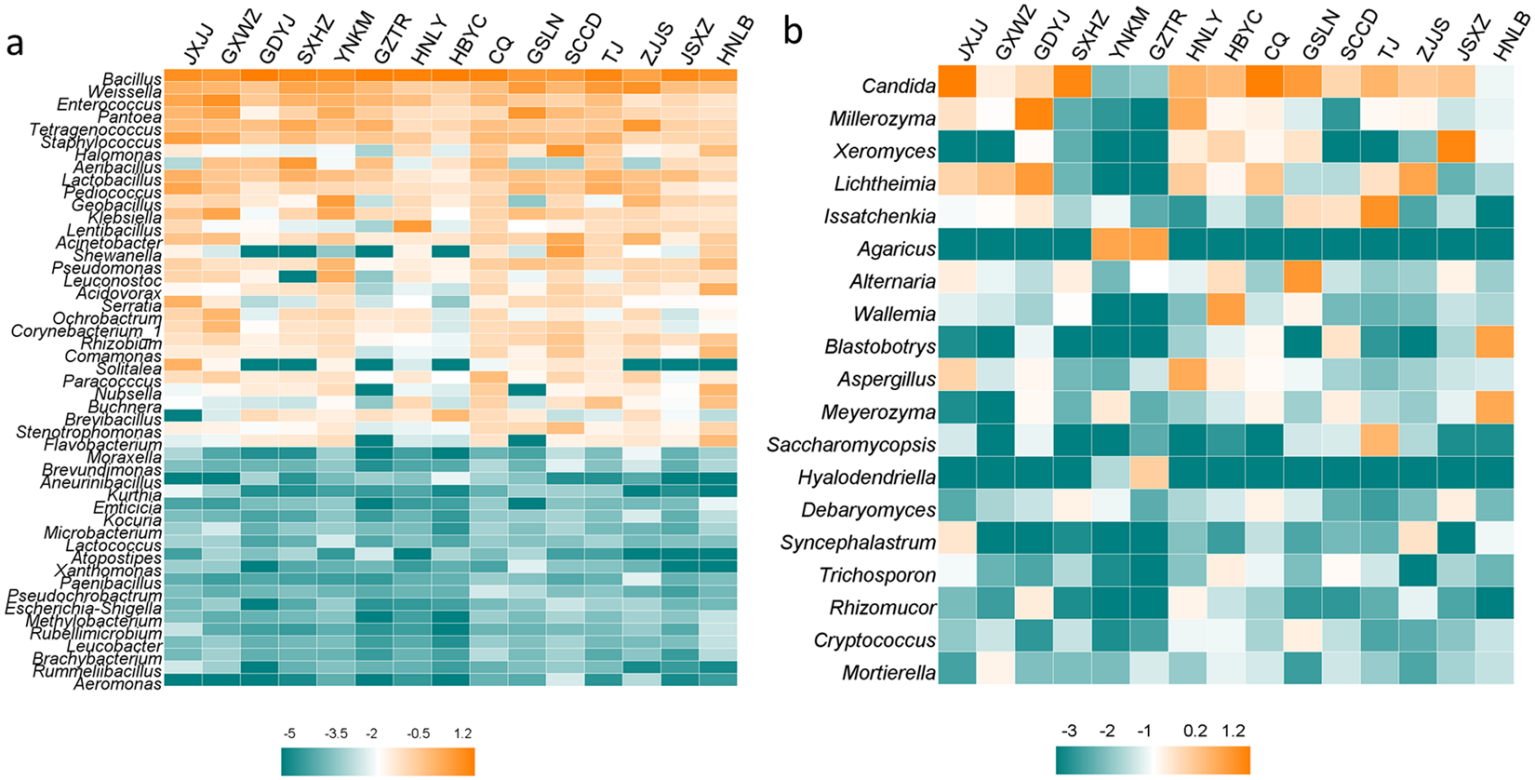
Heatmap of Douchi samples at the genus level. The color intensity of each panel is proportional to the OTU abundance. (**a**) Bacteria, (**b**) Fungi.

Figure 1b showed the difference in fungal abundance among samples at phylum level. *Ascomycota* was the most popular phylum in twelve Douchi samples, and this result was similar to previous studies^16,44^. The heatmap revealed differences in fungal genera among different Douchi samples (Fig. 2b). *Candida* was the most prevailing genus in JXJJ, SXHZ and CQ Douchi samples. Zhang *et al*. reported *Candida* was the dominant genus in Douchi^44^. During Douchi fermentation, *Candida* species grew more quickly than other fungi under high contents of organic acids and salts, then became the dominant fungal species^46^. Additionally, the genera of *Millerozyma*, *Xeromyces*, *Lichtheimia*, *Issatchenkia*, *Agaricus*, *Alternaria*, *Wallemia* and *Blastobotrys* were dominant in some Douchi samples. In a previous study, *Lichtheimia* was also detected as the major genus in Douchi^16^. Garcia *et al*. found that *Lichtheimia* produced β-glucosidase during solid-state fermentation^47^, which contributed to the release of glucose to improve the flavour of Douchi.

### Microbial contribution to BAs contents in Douchi

According to the results of microbial diversity in Douchi samples, the JXJJ, GXWZ, GDYJ, SXHZ, GZTR, HNLY and HBYC Douchi samples were used to isolate strains. Nineteen strains were isolated, named HB-1, HN-1, HN-2, HN-3, HN-4, GD-1, GD-2, GD-3, GD-4, SX-1, SX-2, GZ-1, GZ-2, GZ-3, JX-1, JX-2, JX-3, JX-4 and GX-1. The 16S rDNA or ITS sequences were analyzed to identify these strains, and sequences similarities of isolated strains with representative strains were shown in Table 3. According to the sequences similarities results, these strains were identified as *Bacillus subtilis* (5 isolates), *Bacillus methylotrophicus* (2 isolates), *Bacillus amyloliquefaciens* (2 isolates), *Bacillus sonorensis* (1 isolate), *Bacillus licheniformis* (4 isolates), *Staphylococcus carnosus* (1 isolate), *Staphylococcus pasteuri* (1 isolate), *Enterococcus faecium* (1 isolate) and *Candida* sp. (2 isolates), respectively (Table 3). Most of these strains belonged to the dominant genera in Douchi samples, and they were often reported in fermented soybean products^17,44,48–52^.

**Table 3:** 16S rDNA and ITS sequences similarities of isolated strains with representative microbe.

Isolates	Closest strains	Identities (%)	Accession No.
HB-1	*B*. *subtilis* CS10	99	MH373531
HN-1	*B*. *licheniformis* L5	99	MH373532
HN-2	*B*. *subtilis* L23	99	MH373533
HN-3	*B*. *sonorensis* SXYC17	99	MH373534
HN-4	*B*. *subtilis* yxw4	99	MH373535
GD-1	*B*. *methylotrophicus* HB25	99	MH373536
GD-2	*B*. *amyloliquefaciens* SXAU001	99	MH373537
GD-3	*B*. *licheniformis* JD19	99	MH373538
GD-4	*B*. *subtilis* NJ1	99	MH373539
SX-1	*B*. *licheniformis* DC3-1	99	MH373540
SX-2	*B*. *subtilis* SBE1	99	MH373541
GZ-1	*B*. *licheniformis* JD18	99	MH373542
GZ-2	*B*. *amyloliquefaciens* Y26	99	MH373543
GZ-3	*B*. *methylotrophicus* HB26	99	MH373544
JX-1	*S*. *carnosus* a23	99	MH445557
JX-2	*S*. *pasteuri* HN-35	99	MH445558
JX-3	*Candida* sp. 4 TMS-2011	98	MH443337
JX-4	*Candida* sp. 4 TMS-2011	98	MH443338
GX-1	*E*. *faecium* RK 204	99	MH445559

To further investigate the microbial contribution to BAs accumulation in Douchi, we evaluated the BAs producing and degradation abilities of these isolated strains. The BAs producing abilities of all isolated strains were detected in medium supplemented with corresponding precursors, and the BAs produced by different strains were shown in Table 4. Most of the isolated strains (79%) could produce putrescine via agmatine, while none could synthesize putrescine from ornithine. Only a few strains could generate histamine, tyramine, tryptamine, β-phenethylamine or cadaverine from corresponding substrates. Thereinto, *B*. *subtilis* HB-1 and *S*. *pasteuri* JX-2 showed no ability to produce six common BAs, while *E*. *faecium* GX-1 produced the highest concentration of total BAs, especially the relative high level of tyramine. In addition, *E*. *faecium* also exhibited the ability of generating β-phenethylamine, which was consistent with a previous study^53^.

**Table 4:** The BA-producing abilities of the isolated strains with corresponding precursor.

Strain	Histamine (Histidine) (mg/L)	Tyramine (Tyrosine) (mg/L)	Tryptamine (Tryptophan) (mg/L)	β-Phenethylamine (Phenylalanine) (mg/L)	Putrescine (Ornithine monohydrochloride) (mg/L)	Putrescine (Agmatine sulfate salt) (mg/L)	Cadaverine (Lysine) (mg/L)	Total Bas (mg/L)
HB-1	ND	ND	ND	ND	ND	ND	ND	ND
HN-1	ND	ND	ND	5.42 ± 0.54 a	ND	3.11 ± 0.23 cd	ND	8.53 ± 0.77 ab
HN-2	ND	ND	ND	ND	ND	8.20 ± 0.08 e	ND	8.20 ± 0.08 ab
HN-3	ND	ND	30.98 ± 4.49 a	ND	ND	1.64 ± 0.06 ab	ND	32.62 ± 4.55 c
HN-4	ND	ND	ND	ND	ND	9.8 ± 0.11 f	ND	9.80 ± 0.11 ab
GD-1	ND	ND	ND	7.11 ± 0.32 a	ND	1.48 ± 0.14 a	ND	8.59 ± 0.46 ab
GD-2	ND	ND	ND	ND	ND	7.88 ± 0.62 e	ND	7.88 ± 0.62 ab
GD-3	ND	ND	59.63 ± 6.39 b	ND	ND	1.72 ± 0.24 ab	ND	61.35 ± 6.61 e
GD-4	ND	ND	ND	ND	ND	3.23 ± 0.16 cd	ND	3.23 ± 0.16 a
SX-1	20.91 ± 1.21 a	ND	24.24 ± 6.02 a	ND	ND	2.44 ± 0.10 bc	ND	47.59 ± 7.33 d
SX-2	ND	ND	ND	ND	ND	8.36 ± 0.13 e	ND	8.36 ± 0.13 ab
GZ-1	17.60 ± 5.96 a	ND	27.21 ± 5.98 a	ND	ND	8.64 ± 1.64 e	ND	53.45 ± 13.58 de
GZ-2	ND	ND	ND	ND	ND	8.04 ± 0.22 e	ND	3.04 ± 0.22 a
GZ-3	ND	ND	ND	ND	ND	8.84 ± 0.52 e	ND	8.84 ± 0.52 ab
JX-1	ND	34.76 ± 3.04 a	120.18 ± 4.29 c	378.42 ± 9.15 c	ND	2.12 ± 0.04 ab	ND	535.48 ± 16.52 g
JX-2	ND	ND	ND	ND	ND	ND	ND	ND
JX-3	ND	364.34 ± 7.15 b	ND	ND	ND	ND	2.86 ± 0.53	367.20 ± 7.68 f
JX-4	14.81 ± 0.80 a	ND	ND	ND	ND	3.42 ± 0.11 d	ND	18.23 ± 0.91 b
GX-1	ND	652.76 ± 11.52 c	ND	40.69 ± 2.38 b	ND	ND	ND	693.69 ± 13.98 i

Data are presented as mean ± SDs of three replicates; ND means “not detected”. Different letters (a, b, c, etc.) indicate significantly different means at P < 0.05 (analysis of variance (ANOVA)).

The degrading abilities of the nineteen isolates were investigated. None of the strains was able to cause a complete disappearance of histamine, tyramine, tryptamine, β-phenethylamine, putrescine or cadaverine under the experimental conditions used (Table 5). Among the 19 strains tested, 53% of those strains were able to degrade histamine, 21% for tyramine, 58% for tryptamine, 84% for β-phenethylamine, 11% for putrescine and 11% for cadaverine. Some strains showed high histamine degradation ability (>20%), including *B*. *licheniformis* GD-3, *B*. *subtilis* GD-4, *B*. *subtilis* SX-2 and *Candida* sp JX-3. Moreover, *Candida* sp JX-3 also exhibited a high ability of degrading β-phenethylamine. Consistently, several *B*. *licheniformis* and *B*. *subtilis* strains were also reported to produce histamine in previous studies^50,54^.

**Table 5:** Profiles of BA-degradation rates of selected strains.

Strain	Histamine (%)	Tyramine (%)	Tryptamine (%)	β-Phenethylamine (%)	Putrescine (%)	Cadaverine (%)
HB-1	0.00	0.00	0.00	8.77 ± 0.95 cde	0.00	0.00
HN-1	0.00	0.00	3.29 ± 0.12 b	10.05 ± 1.13 de	0.00	0.00
HN-2	0.00	0.00	0.00	9.98 ± 0.38 de	0.00	1.93 ± 0.12
HN-3	4.42 ± 0.64 a	7.29 ± 0.42 b	2.52 ± 0.20 b	14.56 ± 1.25 f	0.00	6.07 ± 0.54
HN-4	0.00	0.00	1.14 ± 0.17 a	5.34 ± 0.96 bc	0.00	0.00
GD-1	0.00	0.00	2.01 ± 0.13 ab	1.43 ± 0.16 a	0.00	0.00
GD-2	0.00	0.00	2.61 ± 0.26 b	5.93 ± 0.86 bc	0.00	0.00
GD-3	50.53 ± 2.64 d	0.00	0.00	0.00	0.00	0.00
GD-4	50.73 ± 4.06 d	2.45 ± 0.12 a	10.45 ± 1.50 d	11.85 ± 1.20 ef	9.78 ± 0.44	0.00
SX-1	0.00	7.95 ± 0.58 b	0.00	3.75 ± 0.40 ab	7.59 ± 0.62	0.00
SX-2	21.99 ± 2.81 c	0.00	0.00	5.82 ± 0.39 bc	0.00	0.00
GZ-1	11.20 ± 1.49 b	11.06 ± 1.22 c	0.00	14.66 ± 0.66 f	0.00	0.00
GZ-2	0.00	0.00	3.00 ± 0.78 b	1.61 ± 0.44 a	0.00	0.00
GZ-3	0.00	0.00	6.92 ± 1.00 c	4.92 ± 0.32 ab	0.00	0.00
JX-1	0.00	0.00	0.00	0.00	0.00	0.00
JX-2	0.00	0.00	0.00	6.92 ± 0.40 bcd	0.00	0.00
JX-3	56.25 ± 5.98 d	0.00	10.77 ± 0.25 d	50.21 ± 4.98 g	0.00	0.00
JX-4	0.00	0.00	12.96 ± 1.34 e	49.03 ± 5.44 g	0.00	0.00
GX-1	0.00	0.00	9.61 ± 0.22 d	0.00	0.00	0.00

Data are presented as mean ± SDs of three replicates. Different letters (a, b, c, etc.) indicate significantly different means at P < 0.05 (analysis of variance (ANOVA)).

### The BAs-controlling capacity of selected strains in fermented soybean model

**Figure 3:**
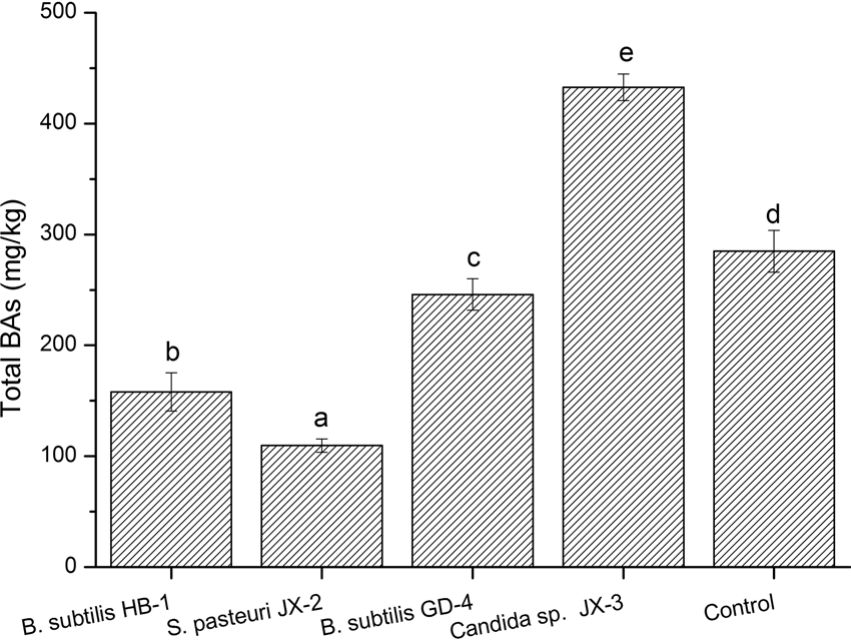
BAs contents in fermented soybean product model inoculated with different strians. Different letters (a, b, c, etc.) indicate significantly different means at P < 0.05.

For the 19 strains, *B*. *subtilis* HB-1 and *S*. *pasteuri* JX-2 showed no BAs producing ability (Table 4), and *B*. *subtilis* GD-4 and *Candida* sp. JX-3 revealed high degradation rates for BAs (Table 5). Those four strains were probably the beneficial candidates for controlling the BAs, and thus their BAs-controlling capacities were evaluated in a fermented soybean model. After fermentation for 7 days, the total BAs content of the control sample without inoculation reached 284.88 mg/Kg (Fig. 3), which might be due to the native microbes in soybean. After inoculation with *B*. *subtilis* HB-1 and *S*. *pasteuri* JX-2, the total BAs concentrations were much lower than that of control (Fig. 3). Meanwhile, the highest BAs content (432.72 mg/kg) was detected in the soybean inoculated with *Candida* sp. JX-3. These results might be due to their different BAs producing ability. Among the four strains, *B*. *subtilis* HB-1 and *S*. *pasteuri* JX-2 had no BAs formation ability, with *Candida* sp. JX-3 exhibiting the highest BAs formation ability (367.2 mg/L). The *B*. *subtilis* HB-1 and *S*. *pasteuri* JX-2 with no BAs producing ability were more beneficial to reduce the BAs accumulation in fermented soybean product, and their existence probably inhibited the growth of BAs-producing strains by competition for nutrients, which might be the main reason for the decreased BAs. Eom *et al*. also observed that *B*. *subtilis* isolated from a Korean traditional fermented food was able to inhibit BA production^50^. In addition, *Staphylococcus xylosus* was used as a starter culture to inhibit BA production in a salted and fermented anchovy^55^. Similarly, the *B*. *subtilis* HB-1 and *S*. *pasteuri* JX-2 isolated in this study can be used as potential candidates for BA control in fermented soybean.

## Conclusion

In summary, the BAs profiles of 15 representative Douchi samples were analyzed and the correlation between microbes and BAs were also investigated. The total BAs contents of all the Douchi samples were within the acceptable dose level, while histamine and β-phenethylamine showed above the toxic level in some samples, indicating that evaluation and control of specific amine are important for Douchi safety. High-throughput sequencing showed that *Bacillus* and *Candida* were the dominant genera. Nineteen strains were isolated from Douchi samples to investigate microbial contribution to BAs accumulation. *B*. *subtilis* HB-1 and *S*. *pasteuri* JX-2 showed no BAs formation ability, and *B*. *subtilis* GD-4 and *Candida* sp. JX-3 revealed high BAs degradation abilities. Moreover, fermented soybean model further confirmed that *B*. *subtilis* HB-1 and *S*. *pasteuri* JX-2 would be the potential candidates for BAs control. This study not only explained the microbial contribution to BAs in Douchi, but also provided potential strains to reduce the BAs in fermented soybean products.

## Electronic supplementary material

Supplementary Dataset 1

## Data Availability

All relevant data are within the paper.
