# Supplementary material for: Biogenic amines analysis and microbial contribution in traditional fermented food of Douchi
Source: Sci Rep. 2018 Aug 22;8:12567. doi: 10.1038/s41598-018-30456-z (PMC6105706; doi:10.1038/s41598-018-30456-z)
Supplement: Supplementary file 1 — Supplementary Dataset 1 [file 41598_2018_30456_MOESM1_ESM.docx]

**Supplementary materials**

**Biogenic amines analysis and** **microbial contribution in traditional fermented food of Douchi**

Lu Li^a,b^, Liying Ruan^a,b^, Anying Ji^a,b^, Zhiyou Wen^a,d^, Shouwen Chen^c^, Ling Wang^a^, Xuetuan Wei^a,b*^

^a^ Key Laboratory of Environment Correlative Dietology (Ministry of Education), College of Food Science and Technology, Huazhong Agricultural University, Wuhan 430070, China

^b^ State Key Laboratory of Agricultural Microbiology, Huazhong Agricultural University, Wuhan 430070, China

^c^ Hubei Collaborative Innovation Center for Green Transformation of Bio-Resources，College of Life Sciences, Hubei University, Wuhan 430062, China

^d^ Department of Food Science and Human Nutrition, Iowa State University, 50011 Ames, IA, USA

**Corresponding author*. Tel./fax.: +86-27-87280670.

*E-mail address*: weixuetuan@mail.hzau.edu.cn

*Postal address*: No. 1 Shizishan Street, Hongshan District, Wuhan 430070, Hubei, P. R. China


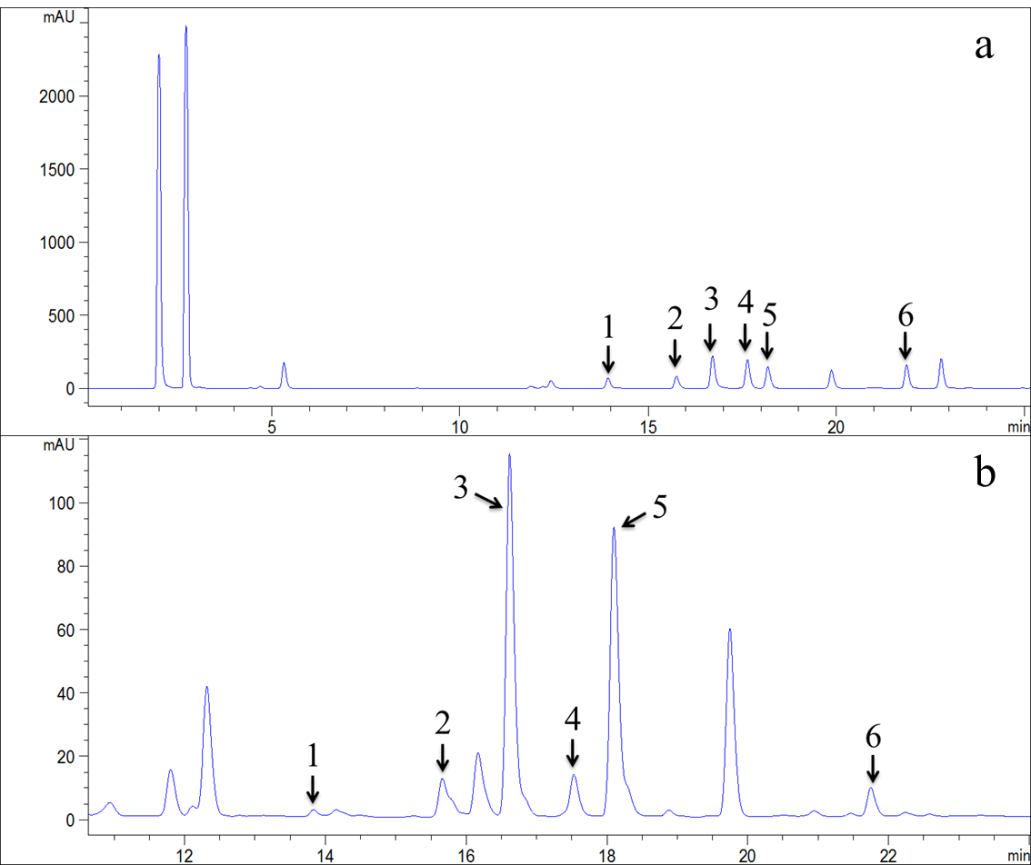


**Figure S1** Typical HPLC chromatograms of BAs in standard solution (a) and in Douchi sample (b). 1, tryptamine; 2, β-phenylethylamine; 3, putrescine; 4, cadaverine; 5, histamine; 6, tyramine.
